# Appendicitis in Bristol—100 Years Ago

**Published:** 1986-12

**Authors:** M J Cooper

**Affiliations:** Consultant Senior Lecturer in Surgery, Department of Surgery, University of Bristol


### Appendicitis in Bristol—100 years ago

**M J Cooper MS FRCS**
Consultant Senior Lecturer in Surgery, Department of Surgery, University of Bristol

In Volume 4 of the Bristol Med-Chi Journal (1886)[1] we find a report of a 13 year old boy who developed abdominal pain in the right iliac fossa. He was anorexic, had vomited and was noted to be pyrexial; examination revealed tenderness localised to the right iliac fossa. Treatment was conservative with turpentine, castor oil and local poultices. When he failed to improve over the next three days, belladonna and laudanum was administered for pain. On the fourth day his pyrexia was resolving but he had a 'tendency to coldness of the extremities'; he died on the morning of the fifth day. A post mortem demonstrated lower abdominal peritonitis and a friable appendix containing a faecalith. The diagnosis made on this unfortunate boy was typhlitis and the treatment prescribed by J. G. Swayne, who later became the Professor of Surgery in Bristol, was standard for this time.

In the same year, 1886, the first meeting of the Association of American Physicians in Washington DC was addressed by Reginald Fitz[2]. His classic paper on 'Perforating inflammation of the vermiform appendix; with special reference to its early diagnosis and treatment' contained the first mention of the word 'appendicitis' and made two important observations. Firstly he emphasised that most inflammatory disease of the right iliac fossa commences in the appendix, and secondly he urged early surgical intervention. Although neither of these points were entirely new, together they delivered a powerful message which the American surgeons were quick to exploit with a rapid fall in the mortality rate from appendicitis. The conservative British surgical establishment was slow to follow and surgery was usually delayed until crepitus was apparent. In this article I will attempt to explain the events that led up to this change in practice.

### ANATOMY

The appendix is clearly visible in the anatomical drawings of Leonardo da Vinci[3] produced late in the 15th century. It was first described in 1521 by Berengoria da Carpi,[4] although it did not receive its name until 1699.[5] By 1719 Morgagni[6] had fully described the variability of its position and little has been added since.

### PATHOLOGY

Appendicitis occurred in the time of the Pharoahs[7], but the first clear account was by Lorenz Heister in 1711.[8] As the Professor of Surgery, he was called upon to perform an autopsy in the public theatre at Altdorf on a recently executed criminal. He found the appendix to be black and perforated, although no relevant history was available. His statement that 'the instance may stand as proof of the possibility of inflammation arising and abscesses forming in the appendicula', was largely ignored for another 150 years. Forty years later Mestivier[9] in Paris described appendicular perforation by a pin and since then a long list of foreign bodies have been found in the appendix.[10] John Hunter described appendicitis in an autopsy on Colonel Dalrymple in 1767,[11] but no further studies followed.

The 19th century was a time of great dissension. In 1824 Louyer-Villermay[12] described two cases of gangrenous appendicitis and encouraged Melier[13] who suggested that surgery might be the answer. However, European thoughts were fossilised by the prevailing view of Baron Dupuytren. He was Chief of Surgery at the Hotel Dieu and was one of the leading teachers and surgeons of his day; he taught that right iliac fossa inflammation was caecal in origin and secondary to the weight of faeces in the right colon.[14] A similar train of thought led Goldbeck[15] to clearly describe 30 cases of appendicitis and then call the disease perityphlitis; Albers[16] in Bonn was able to separately classify four different types.

Although several British authors[17,18] were to mention appendicitis in the early half of the century, laparotomy was still a perilous procedure and the implications unclear. Fitz altered all this with a convincing message at a time when anaesthesia and antisepsis had made things much safer. By 1894, when the previously mentioned Mr Swayne wrote again in the journal,[19] appendicitis was noted to be a common disease due to a foreign body. Exactly how common it was is unknown. Rendle Short[20] went through the notes of admissions to the Bristol Royal Infirmary between 1880 and 1918. He could only find 4 probable cases in 1880 rising to 113 in 1918; the number of inpatients treated in this period only rose 55% so this was truly a massive increase. This was a careful survey in which he also considered the effects of private practice and the fact that patients might not have made it to hospital; there were apparently plenty of empty beds in those days! Treves[21] however in 1902 clearly thought that the disease was not new, but had merely passed under a variety of other names. The answer lies somewhere between the two opinions, but there is evidence that appendicitis is a 20th century phenomenon which is now past its peak.[22]

Invasion of the appendicular wall by locally resident bacteria is the ultimate event secondary to luminal obstruction due to lymphoid hyperplasia or a faecalith. Many of these factors were worked out as far back as 1905.[23] Rendle Short[20] in a careful dietary survey concluded that the increase in cases was due to a reduction in ingested cellulose; certainly in accordance with modern theory.

### CLINICAL FEATURES

Appendicitis can occur at any age and remains an essentially clinical diagnosis. The signs and symptoms are well known and no blood or radiological investigation has proven of sufficient value to be of use in all but the occasional case. Fear of perforation has led to a high negative laparotomy rate, but the advent of antibiotics has reduced the dangers of peritonitis and a period of careful observation is warranted if there is any doubt. Some authors advocate laparoscopy, but most surgeons will agree with Way et al[24] that 'considered as a diagnostic test, appendectomy is probably more cost-effective and, certainly more reliable than laparoscopy'.

### TREATMENT

With very few exceptions the treatment of acute appendicitis is appendicectomy. The operation was first performed in London in December 1735.[25] Claudius Amyand, a surgeon at St George's Hospital, explored an inguino-scrotal hernia in an 11 year old boy, who had recently developed a faecal fistula. On exploration he found that the appendix was perforated by a pin; appendicectomy was followed by recovery. Little else was to happen for nearly 150 years although in 1838 Stokes recommended opium for peritoneal inflammation; the main benefit of this treatment was that it permitted a peaceful death.

Hancock[26] in London reported draining an appendix abscess in 1848; the wound discharged for some time, and a faecalith was expelled as the patient recovered. In the United States Parker[27] advocated opening abscesses at the early stage of 5–12 days into the illness and reduced his mortality from 50% to 15%. Lawson Tait[28] actually performed a curative appendicectomy in 1880, but this was not reported until 10 years later, by which time he had abandoned the procedure as unnecessarily risky! Hall[29] performed the first appendicectomy (appendectomy) in America through a hernia in 1886. Under the guidance of Parker[27] and Fitz[2] American surgeons came to believe in early appendicectomy and escaped from the dogma of Dupuytren. Parker's pupil, Sands,[30] developed an interest which he passed on to McBurney who wrote extensively on the disease and its treatment[31].

Many famous people have suffered from appendicitis. In 1897 Harvey Cushing, then a surgical resident, diagnosed the condition on himself and with some difficulty persuaded William Halstead to operate within 24 hours; he made an eventful recovery.[32] It was the illness of King Edward VII in 1902 that made appendicitis fashionable and changed the treatment in Britain.[33] On June 13 only 12 days before his coronation, Edward complained of abdominal pain. Five days later he was seen by Frederick Treves who had performed his first appendicectomy in 1888 and had developed an interest in the disease. Treves elected to wait with the British Medical Journal reporting that Edward had appendicitis, whilst the Lancet considered the term barbaric and described it as perityphlitis. Two days before the coronation Edward was improved enough to hold an eight course banquet. He relapsed that night and Treves persuaded him to undergo an operation; he recovered well and was crowned in August. Treves later saw his own daughter die from unoperated acute appendicitis. George Fowler who advocated the Fowler position in some cases of peritonitis, died after an operation for a perforated appendix in 1906.

The operative technique is well known with most surgeons using the approach described by McBurney in 1894[31]. Management of the appendix stump has been controversial over the years, but most modern surgeons ligate the appendix before amputation and use a purse string to bury the stump; in 1905 Kelly[34] felt that both these manoeuvres were likely to cause trouble.

During the first half of the century many authors published large series of appendicectomies largely for 'chronic' appendicitis; in the opinion of some surgeons largely for financial gain. Wth the introduction of antibiotics and later their prophylactic use,the morbidity rate has dropped to a low level.

### APPENDIX MASS AND INTERVAL APPENDICECTOMY

By 1900 early surgery was the norm in the USA, but it was recognised that some patients still presented after perforation. Ochsner recommended in 1902[35] that these patients should be treated conservatively, permitting the paralytic ileus to wall-off the infection. Scherren[36] supported him and with careful care the mortality dropped considerably. With the introduction of antibiotics Fields[37] encouraged early intervention, but it now seems that most patients do well either way. The concept of interval appendicectomy was introduced by Murphy in 1904[38] and has been routine ever since to prevent recurrent appendicitis. It now seems that this risk has been overstated, is probably only 10–20%, and that appendicectomy is certainly not warrranted in the elderly or unfit[39].

### MORTALITY

Death from appendicitis is now almost confined to the extremes of life, for it is in these patients that perforation occurs early; overall mortality is now around 0.1%. Berry and Malt[40] have recently reviewed 72 of Fitz's initial cases and compared them with a modern group of 300 patients. In 1890 the overall mortality was 26%. Half Fitz's patients were treated surgically with a 40% mortality; observation was safer with a death rate of 11%. By 1980 the mortality rate had dropped to 0.3% but 25% of patients were still presenting after perforation.

We can only guess at the mortality rate in Bristol 100 years ago since appendicitis did not become a separately notifiable and recorded cause of death until 1901; there were then 38 deaths per million living per annum. Treves[21] put his case mortality at around 20% and Morton[41] reviewing his last 40 cases of appendicitis in the Journal in 1900 noted that 10 died (25%). Morton[42] wrote again in 1905 when he reported 155 cases in total with 30 deaths (19%) so that some improvement had occurred. By the 1920s appendicectomy prior to perforation was a relatively safe operation with a death rate of 0.9%; once peritonitis ensued the rate rose to 20%. Large series from the USA included many operations for chronic appendicitis; Guerry[43] reported 1,241. The big decline occurred between 1940 and 1960, and although this coincides with the development of antibiotics, Truelsen believes it to be the result of improved fluid and electrolyte management.

### CONCLUSION

The appendix is a small organ which in man is of no known benefit. Throughout the years it has caused untold misery and although death has become much less common since Fitz's paper 100 years ago, presentation is still delayed until after perforation in 25% of patients. Only by achieving earlier presentation will the morbidity and mortality further improve.

### REFERENCES

1. SWAYNE, J. G. Case of typhlitis. Br. Med. Chi. J. 1886, 4; 108–14.
2. FITZ, R. H., Perforating inflammation of the vermiform appendix. Am. J. Med. Sci. 1886, 92; 321–46.
3. McMURRICH, J. P. Leonardo Da Vinci The Anatomist. 291, Williams and Wilkins, Baltimore 1930.
4. Da CARPI, B. Commentaria (1521).
5. VERHEGEN. Corporis humani anatomia (1699)
6. MORGAGNI, G. B. Adversaria anatomica tertia 24–28 (1719).
7. BETT, W. R. Appendicitis. In: Bett, W. R. (ed), A short history of some common diseases, 162–171, Oxford University Press, London, 1934.

8. MAJOR, R. H. Classic descriptions of disease. 648–650. C. C. Thomas, Sprinfield, Ill. 1965.
9. MESTIVIER, M. Observation sur une tumeur situee pres de la region ombilicale, du cote droit, occasionee par une grosse epingle trouvee dans l'appendice vermiculaire du caecum. J. Med. Clin. Pharm., Paris 1759 10; 441–442.
10. BALCH, C. M. and Silver D. Foreign bodies in the appendix. Arch. Surg. 1971; 102; 14–20.
11. HUNTER, J. In: Cope Z. A history of the acute abdomen, Oxford University Press, London 1965, p. 20.
12. LOUYER-VILLERMAY, J. B. Observations pour servir a l'histoire des inflammations de l'appendice du caecum. Archs. Gen. Med., Paris 1824; 5: 246–250.
13. MELIER, F. Memoires et observations sur quelques maladies de l'appendice cecal. J. Med. Chir. Pharm., Paris 1827, 317–345.
14. DUPUYTREN, G. Des abces de la fossa iliaque droite. Lecons Chir. 1833, 3; 330–332.
15. GOLDBECK, G. Uber eigenthumliche entzundliche Geschwulste in der rechten Huftbeingegend. Kranzbuiher, J. A., Worms 1830.
16. ALBERS, J. F. H. Geschichte de Blindarmentzundung. Beobgeb Path. Anat. 1837, 2; 1–37.
17. BURNE, J. Of inflammation, chronic disease, and perforative ulceration of the caecum and of the appendix vermiforms, with symptomatic peritonitis and abscess. Med. Chir. Trans. 1837, 20; 200–229.
18. BRIGHT, R. and ADDISON, T. Elements of the Practice of Medicine. Longmans Green and Co, London 1839.
19. SWAIN J. Appendicitis. Br. Med. Chi. J. 1894, 12; 9–25.
20. SHORT, A. R. The causation of appendicitis. Brit. J. Surg. 1920; 171–188.
21. TREVES, F. Some phases of inflammation of the appendix. Br. Med. J. 1902, 1; 1589–1594.
22. NOER, T. Decreasing incidence of acute appendicitis. Acta. Chir. Scand. 1976, 141; 431–432.
23. BOTTOMLEY, F. C. The causes of appendicitis. Practitioner. 1905, 74; 827–832.
24. WAY, C. W. V., Murphy, J. R., Dunn, E. L. and Elerding, S. C. A feasibility study of compurer aided diagnosis in appendicitis. Surg. Gynecol. Obstet. 1982, 155; 685–688.
25. AMYAND C. Of an inguinal rupture, with a pin in the appendix caeci, incrusted with stone; and some observations on wounds in the guts. Philos. Trans. R. Soc., London 1736, 39; 329–342.
26. HANCOCK, H. Disease of the appendix caeci cured by operation. Lancet 1848, 2; 380–381.
27. PARKER, W. An operation for abscess of the appendix veriformis caeci. Med. Rec. New York 1867, 2; 25–27.
28. TAIT, L. The surgical treatment of typhlitis. Birmingham Med. Rev. 1890, 27; 26–34 and 76–89.
29. HALL, R. J. Suppurative peritonitis due to ulceration and supuration of the vermiform appendix. New York Med. J. 1886, 43; 662–663.
30. SANDS, H. B. An account of a case in which recovery took place after laparotomy had been performed for septic peritonitis due to a perforation of the vermiform appendix with remarks upon this and allied diseases. New York Med. J. 1888, 47; 197–205.
31. McBURNEY, C. The incision made in the abdominal wall in cases of appendicitis, with a description of a new method of operating. Ann. Surg. 1894, 20; 38–43.
32. FUILTON, J. F. Harvey Cushing, A Biography. Springfield, C. C. Thomas, 1946.
33. STEVENSON, S. R. In: Famous illnesses in history. 32–43 Eyre, London 1962.
34. KELLY, H. A. In: The vermiform appendix and its diseases. W. B. Saunders and Co, Philadelphia 1905.
35. OCHSNER, A. J. In: A handbook of appendicitis. G. P. Gerhardt, Chicago 1902.
36. SHERREN, J. The causation and treatment of appendicitis. Practitioner 1905, 74; 833–844.
37. FIELDS, I. A., Naiditch, M. J. and Rothman, P. E. Acute appendicitis in infants. Am. J. Dis. Child 1957, 93; 287–301.
38. MURPHY, J. B. Two thousand operations for appendicitis with deductions from his personal experience. Am. J. Med. Sci. 1904, 128; 187–197.
39. BRADLEY, E. L. and ISAACS, J. Appendiceal abscess revisited. Arch. Surg. 1978, 113; 130–132.
40. BERRY, J. and MALT, R. A. Appendicitis near its centenary. Ann. Surg. 1984, 200; 567–575.
41. MORTON, C. A. Forty cases of operation for appendicitis. Br. Med. Chi. J. 1900, 18; 320–330.
42. MORTON C. A. A study of the records of one hundred and fifty-five cases of operation for appendicitis. Br. Med. Chi. J. 1905, 23; 223–267.
43. GUERRY, L. G. A study of mortality in appendicitis. Ann. Surg. 1926, 84; 283–287.

